# Total laparoscopic radical antegrade modular pancreato-splenectomy with left-posterior superior mesenteric artery first-approach for distal pancreatic cancer: step-by-step technique with a surgical case report (with video)

**DOI:** 10.1186/s12957-022-02657-4

**Published:** 2022-07-05

**Authors:** Thanh Khiem Nguyen, Ham Hoi Nguyen, Tuan Hiep Luong, Kim Khue Dang, Van Duy Le, Hong Son Trinh, Duc Dung Tran

**Affiliations:** 1 Department of Gastrointestinal and Hepato-pancreato-biliary surgery, Bach Mai Hospital, Hanoi, Vietnam; 2 Department of Surgery, Hanoi Medical University, 1st Ton That Tung Street, Dong Da, Ha Noi 11521 Vietnam; 3 Department of Oncology, Viet Duc University Hospital, Hanoi, Vietnam; 4 Department of Surgery, Thai Binh Medical University, Thai Binh, Vietnam

**Keywords:** Total laparoscopic, Radical antegrade modular pancreato-splenectomy, Left posterior SMA first-approach, Case report

## Abstract

**Introduction:**

Left-sided pancreatic cancers are uncommon but seem more aggressive than tumors of pancreatic head. Radical antegrade modular pancreato-splenectomy (RAMPS) was proved to have safe and effective advantages while comparing with standard retrograde pancreato-splenectomy (SRPS) in recent systematic literature reviews and meta-analyses. Laparoscopic SMA first-approach RAMPS was recently proceeded with optimistic perioperative outcomes.

**Case presentation:**

Our patient is a 67-year-old female with a medical history of diabetes and hypertension, recruited because of upper left quadrant abdominal pain. She was referred for pancreato-splenectomy because of a 3-cm-sized mass in distal pancreas. We use 5 trocars and the patient placed in a Trendelenburg position. The retroperitoneum is opened at the left-posterior side of the meso-pancreato-duodenum along to the inframesocolic space, so that the anterior surface of the aorta (AO), inferior vena cave (IVC), left renal vein (LRV), left adrenal grand (LAG), and kidney are completely exposed. The inferior border of the pancreas had been dissected and separated from the superior mesenteric vein (SMV) below the pancreatic isthmus, removed the lymph nodes (LNs) groups 14v and 17. Then, dissect of LNs groups 7,8,9,11p,12 en bloc at the superior side of the pancreas. Dissection of LNs group 14p, d or SMA LNs after transecting the pancreas. The operation time was 240 min, the estimated blood loss was 200 ml. With no postoperative complications as well as no diarrhea, the patient was discharged on the POD10 uneventfully. Pathological result: pancreatic ductal adenocarcinoma with T2N1 staging and negative margin (R0).

**Conclusions:**

This technique was safe and effective to perform precise and complete lymphadenectomy and negative posterior resection in total laparoscopic left-posterior SMA first-approach RAMPS for distal pancreatic cancer.

**Supplementary Information:**

The online version contains supplementary material available at 10.1186/s12957-022-02657-4.

## Introduction

Pancreatic ductal adenocarcinoma (PDAC) is one of the most malignant gastrointestinal cancers. Among them, tumors of the pancreatic body and tail, or left-sided PDAC are uncommon, which accounted for about one-third of all the pancreatic neoplasms [1], and supposedly seem more aggressive than tumors of pancreatic head, not only cause by the late presentation, but also due to the difference of molecular characteristics, embryology and histology and therapeutic response [2]. Surgery is considered the only curative treatment method in pancreatic cancer, and Radical antegrade modular pancreato-splenectomy (RAMPS), which was firstly published by Strasberg et al., was proved to be superior in safe and effective aspects while comparing with standard retrograde pancreato-splenectomy (SRPS) in recent systematic literature reviews and meta-analyses [3–5]. Otherwise, SMA first-approach has been proved with benefits of improving R0-resection rate and better perioperative outcomes, as well as better long-term survival particularly [6]. Recently, with the development of laparoscopic instruments and techniques, total laparoscopic SMA first-approach RAMPS was recently proceeded by some surgeons with optimistic perioperative outcomes versus open SMA first-approach RAMPS [7, 8].

With experiment of total laparoscopic SMA first-approach pancreaticoduodenectomy (PD), herein we present a novel technique called total laparoscopic left-posterior SMA first-approach RAMPS, with demonstrated on describing the procedure step by step with illustrative images and video (Additional file 1). All our work has been reported in line with the CARE criteria and guidelines [9].

## Case presentation

Our patient is a 67-year-old female with a medical history of diabetes and hypertension, recruited because of upper left quadrant abdominal pain. She was referred for pancreato-splenectomy because of a 3-cm-sized mass in distal pancreas. The operation time was 240 min, the estimated blood loss was 200 ml. With no postoperative complications as well as no diarrhea, the patient was discharged on the POD8 uneventfully. Pathological result: pancreatic ductal adenocarcinoma with T2N1 staging and negative margin (R0).

The reported case of a 67-year-old female patient a medical history of diabetes and hypertension. She admitted to the hospital because of upper left quadrant abdominal pain. There were no significant findings on physical examination with the exception of mild malnutrition. Height was 150 cm and weight were 50 kg. Laboratory findings were as follows: total bilirubin, direct bilirubin, and albumin was within normal range, and amylase was 682 U/l (the normal range was 28–100 U/l). The serum level of carbohydrate antigen (CA)19-9 was 862 U/mL, and carcinoembryonic antigen (CEA) was 3.72 ng/ml. Abdominal contrast-enhanced computed tomography (CT) scan revealed a 30-mm solid mass in distal pancreas. There was no evidence of lymph node metastasis, peritoneal dissemination, or distant organ metastasis. The final diagnosis was an adenocarcinoma tumor of the Ampulla of Vater with TNM Staging was cT2N0Mx according to The American Joint Committee on Cancer (AJCC) 8th Staging [10]. MDT meeting consisted of surgeons, physicians, clinical and medical oncologists, radiologists, pathologists, and clinical nurse specialists (CNSs) were organized to make clinical decisions. The informed consent was signed, and RAMPS technique was performed.

### Surgical technique

We use five trocars: one 10 mm-trocar placed through the umbilicus for camera; two 12 mm-trocars placed at the midclavicular line higher 1 cm compared to umbilicus in the right and left side for instrument; two 5 mm-trocars placed at right and left subcostal, and the patient placed in a Trendelenburg position. Surgeon stands on the right side of patient in the SMA’s dissection phase, and changes to the middle position when dissecting the posterior surface of the artery with the rest of the surgical phases, the second and third assistants holding the middle and right cameras, the first assistant standing on the right side.

**Fig. 1:**
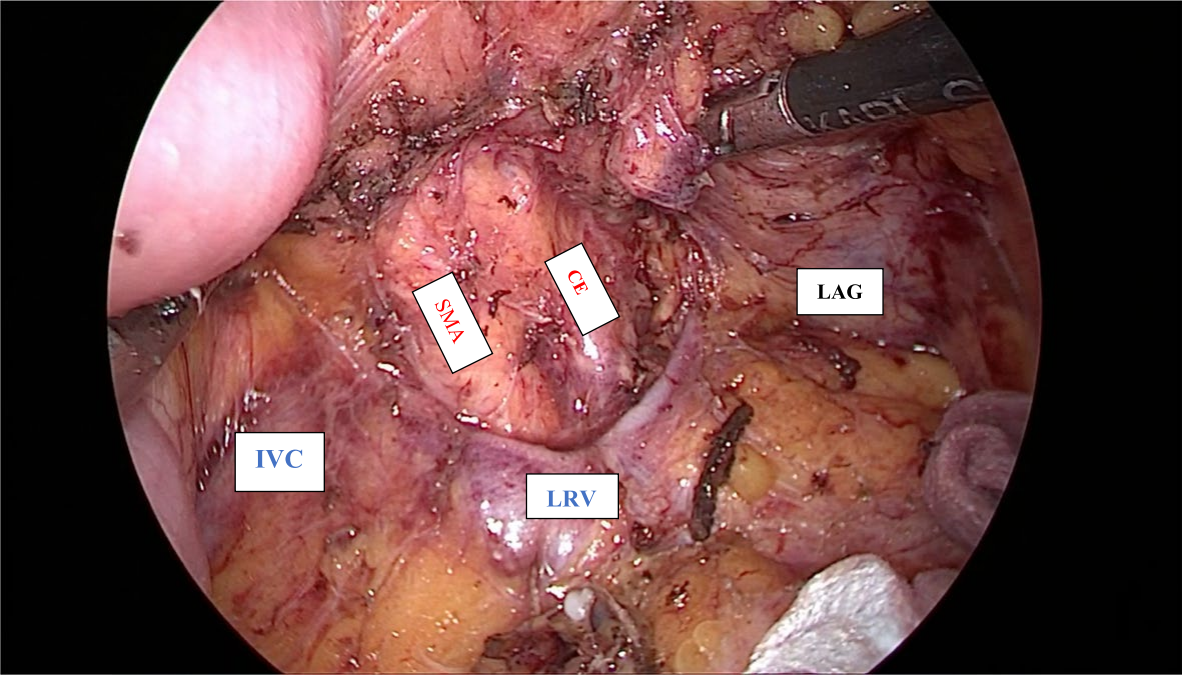
The retroperitoneum is opened at the left-posterior side of the meso-pancreato-duodenum to excluding posterior surgical invasion (SMA: superior mesenteric artery, CE: celiac axis, IVC: inferior vena cave, LRV: left renal vein, LAG: left adrenal grand)

**Fig. 2:**
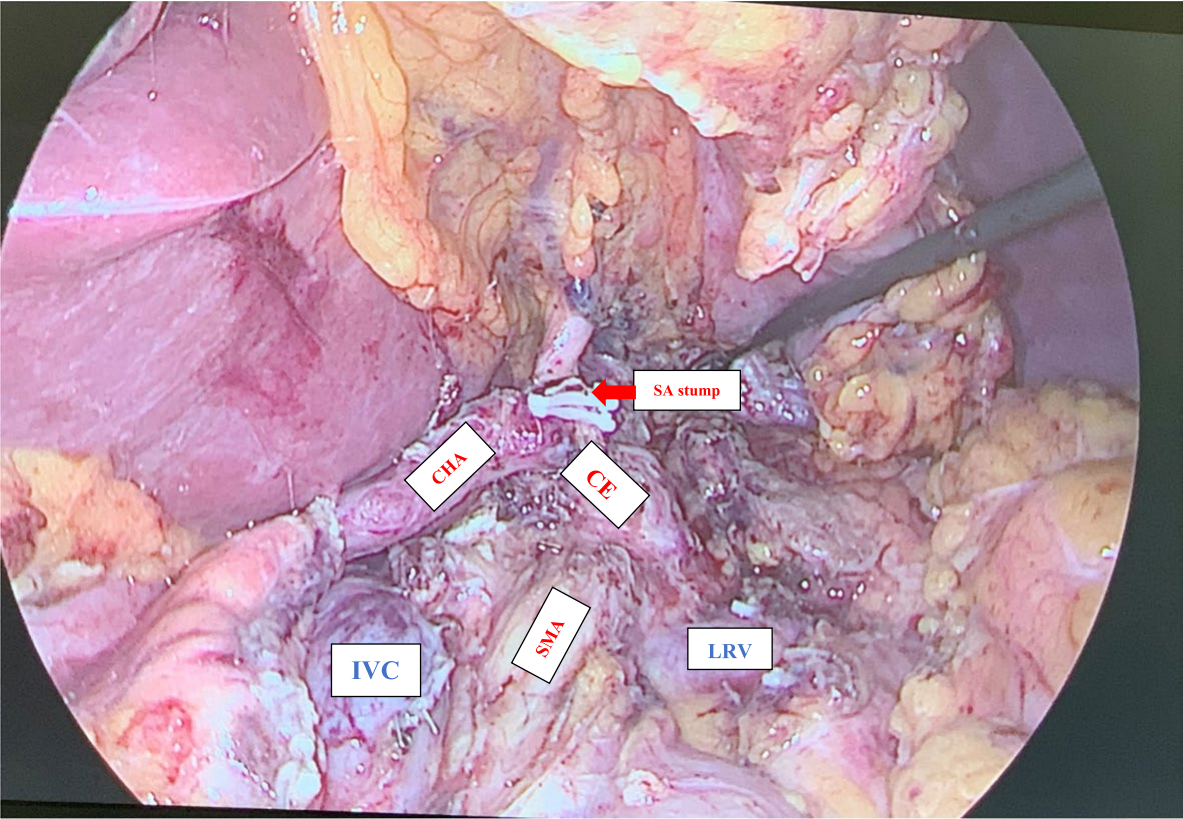
Dissection of LNs groups 7,8,9,11p,12 en bloc and LNs group 14p, d or SMA LNs with preserving the nerve plexus around the SMA (pl-SMA) and celiac axis (pl-CE) (SMA: superior mesenteric artery, CE: celiac axis, CHA: common hepatic artery, SA: splenic artery, IVC: inferior vena cava, LRV: left renal vein)

**Fig. 3:**
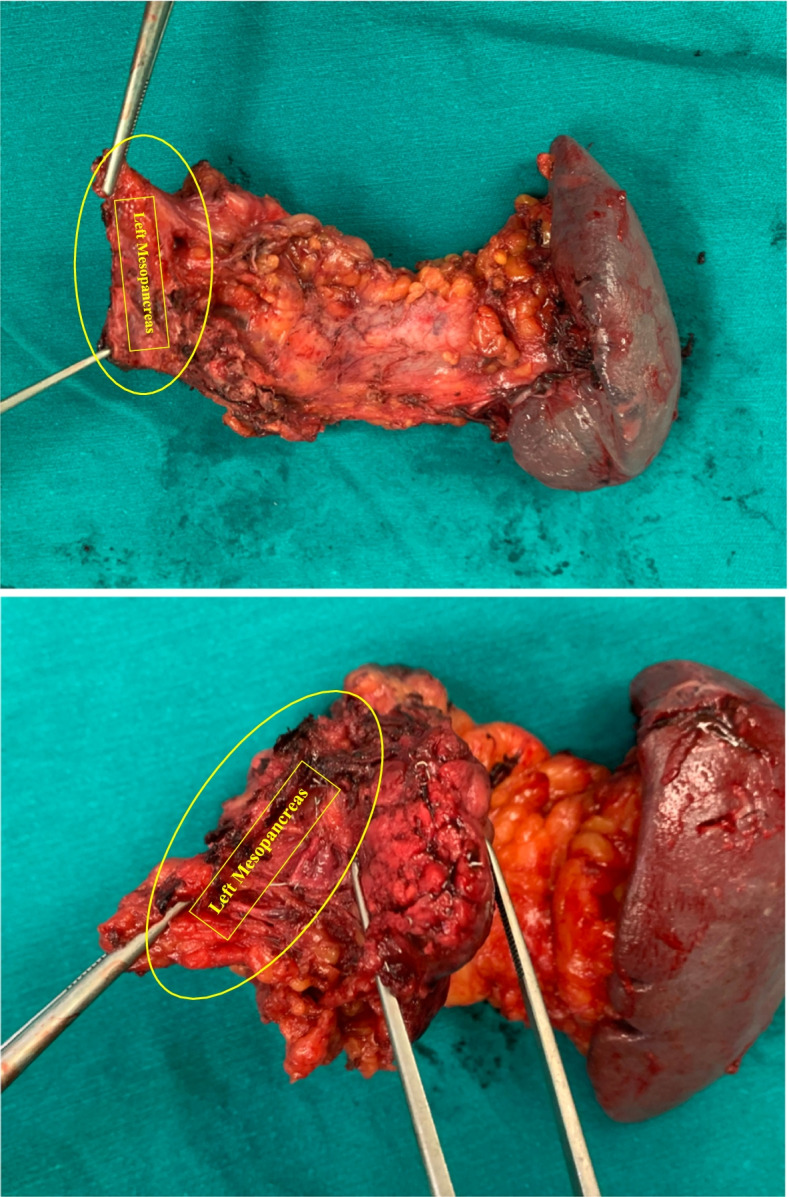
Specimen with the left meso-pancreas, or ‘lame rétroportale gauche’

**Fig. 4:**
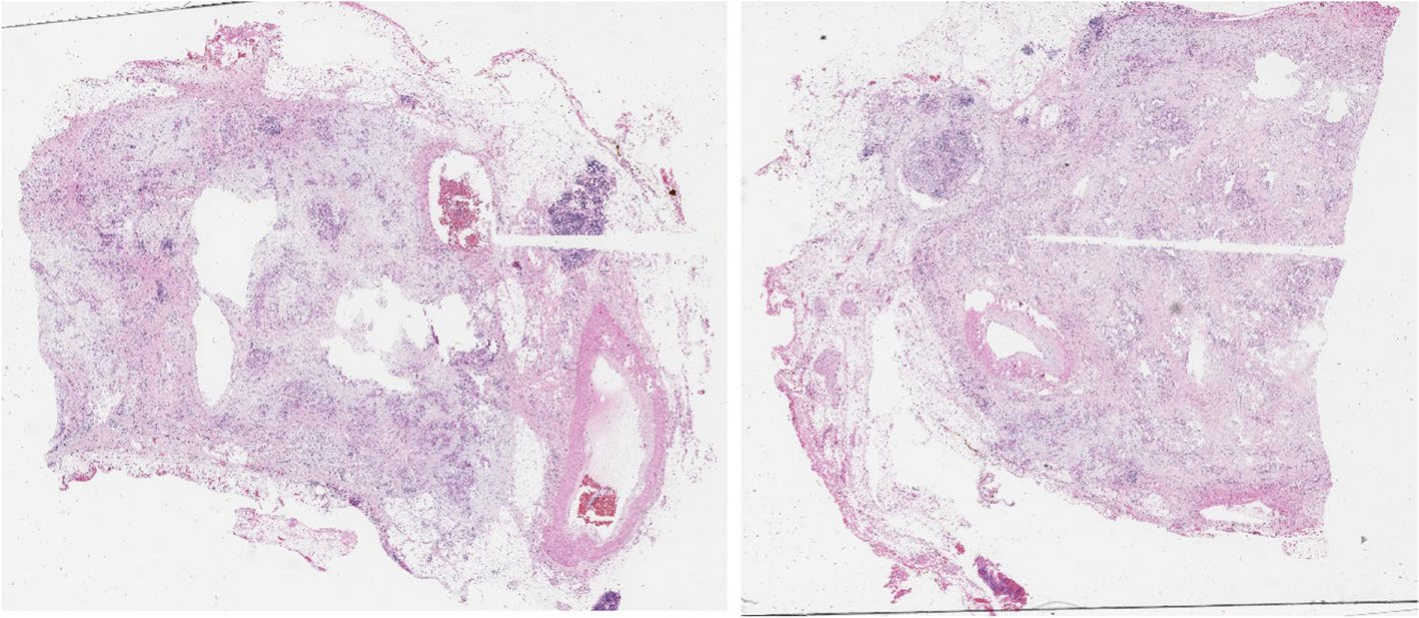
The dorsal margin of the tumor using cross section of specimen in our patient without evidence of tumor invasion

After exploring the peritoneum to exclude metastases, the left-sided assistant lifts the mesentery of transverse colon upward, and the right-sided assistant pulls the first jejunum to the right side. The retroperitoneum is opened at the left-posterior side of the meso-pancreato-duodenum along to the inframesocolic space, so that the anterior surface of the aorta (AO), inferior vena cave (IVC), left renal vein (LRV), left adrenal grand (LAG), and kidney are completely exposed, as well as the posterior side of pancreatic body to excluding posterior surgical invasion (Fig. 1). The anterior or posterior RAMPS were performed depending on whether the tumor infiltrated to the LAG or not [3]. Then, the gastrocolic and gastrosplenic ligaments are divided, and the inferior border of the pancreas had been dissected and separated from the superior mesenteric vein (SMV) below the pancreatic isthmus, removed the lymph nodes (LNs) groups 14v and 17. Then, go upward to the superior side of the pancreas to dissect of LNs groups 7,8,9,11p,12 en bloc. After transecting the pancreas, carefully dissect the distal pancreas with the SMA, including dissection of LNs group 14p, d or SMA LNs with preserving the nerve plexus around the SMA (pl-SMA) and celiac axis (pl-CE) (Fig. 2). Suture and cut the pancreatic isthmus, find and suture Wirsung’s tube with Prolen 5/0 X-stitch, and suture U-stitch with Prolen 4/0, or just using a Surgical Stapler^TM^. Then continuedly expose left gastric artery (LGA), common hepatic artery (CHA) and splenic artery (SA) then ligate the SA just close its stump. Then the splenic vein (SV) is revealed at the splenic-mesenteric confluence, ligation of the splenic vein at the root. Left meso-pancreas, or ‘lame rétroportale gauche ’[11], a retro-pancreatic structure connected the left-side SMA with distal pancreas was dissected en bloc with specimen (Fig. 3). The dorsal margin of the tumor using cross section of specimen in our patient showed without evidence of tumor invasion (Fig. 4).

## Results

The operation time was 240 min, the estimated blood loss was 200 ml. With no postoperative complications as well as no diarrhea, the patient was discharged on the POD10 uneventfully. Pathological result: Pancreatic ductal adenocarcinoma with T2N1 staging and negative margin (R0).

## Discussion and conclusion

Tumors, especially PDAC, of distal pancreas are still considered a group with poor prognosis compared to other gastrointestinal cancers. According to GLOBOCAN 2020, every year in the world there are nearly 496,000 new cases and more than 466,000 deaths [12]. It can be explained that the symptoms are atypical, so patients often admitted to the hospital with advanced stage, although there have been many advances in diagnostic tools such as computed tomography (CT) scan, tumor markers, biopsies under endoscopic ultrasound (EUS) etc. [2]. Distal pancreatectomy was first proposed by the Mayo Clinic in 1913 to treat tumors of the pancreatic body and tail and is still widely used today. However, the 5-year survival rate is still low (6–30%), the median survival is 1–28 months [1]. The reason is mostly explained that patients were diagnosed when they are at a late stage, the rate of R0 resection is still low (77–87%) and the rate of lymph node metastasis is high [2, 13, 14]. So, the most important thing, in terms of surgery, is necessary to remove the tumor with extensive lymph node dissection, ensuring a negative resection area.

Surgery is considered the only curative treatment method in pancreatic cancer, with extensive resection of the tumor and lymph node dissection ensuring a negative resection are important in improving the prognosis of pancreatic tumors [15]. The rate of surgical indications at time of diagnosis is only 10–20% and the prognosis after surgery is still limited [16]. In 2003, Strasberg first published the RAMPS technique. Based on the characteristics of lymph node metastasis, RAMPS was developed based on the standard distal pancreatectomy technique, with technological changes to lymph node dissection and posterior dissection ensure a negative resection and control bleeding. In this technique, Strasberg performed dissection of the LNs group N1 (including splenic hilar, spleen-gastric, inferior pancreatic, and gastroduodenal lymph nodes) and group N2 (LNs of celiac artery (CA) and SMA). The results of this technique were performed and evaluated on 10 patients; the study results showed that up to 9 patients reached R0 (accounting for 90%). The proper RAMPS technique guaranteed two objects: extensive lymph node dissection (dissection of the LNs around CA and hepatic hilum, in this phase can ligate the LGA to help perform dissection easier if the patient is obese, ligation of the LGV. Continue to remove longitudinally the LNs of the hepatic artery going down and to the left side, open the transverse peritoneum at the base of the caudal lobe, remove all fat tissue and LNs around the CA. Expose and completely ligate the SA) and negative margin (R0) on the left and posterior sides. With the left side, it’s required to remove all connective tissue along the left border of SMA to its origin from the abdominal aorta, the LNs located anterior to the aorta between the SMA, and the CA should be removed; so the left margin should clearly see the origin of the CA and SMA arising from the aorta. For the posterior side, there are 2 options that can go before or after the left adrenal gland, depending on whether an infiltration of the tumor to the left adrenal gland is suspected or not [3]. Moreover, in our technique, we have isolated the celiac trunk posteriorly (Fig. 1) that have not mentioned in Ome’s report yet [17]. We think this maneuver help totally control the posterior margin as well as support to go upward the superior side of the pancreas to dissect of LNs groups 7,8,9,11p,12 en bloc.

Detecting the status of tumor infiltration to the SMA is one of the most important factors that determined the resectability of PDAC tumor according to Guidelines of National Comprehensive Cancer Network (NCCN) as well as Japanese Pancreas Society (JPS) [18, 19]. Like PD, “SMA-first approach” means detecting the state of tumoral invasion of superior mesenteric vessels as well as celiac trunk and portal vein to determine the resectable conditions before the point of no return (the step of pancreatic dissection or the splenic vessels division) [7]. We use left-posterior SMA-first approach for two reasons. Firstly, this approach had been proved with superior advantage in achieving a negative resection margin (R0) around the major vessels [6, 20]. Secondly, due to the left renal vein (LRV) has been identified as an important landmark to enter the appropriate posterior layer in RAMPS, left-posterior SMA-first approach was recommended to ensure entry into the dorsal dissecting layer of the pancreas [17, 21]. Laparoscopic left-posterior SMA-first approach RAMPS had just been developed in recent years, with some case reports has been published [7, 22]. An original article by Kawabata et al. in comparing laparoscopic versus open SMA-first approach RAMPS has shown the similarities of two procedures in the overall morbidity, as well as the total number of dissected and harvested lymph nodes around the SMA and R0 resection rates, but significantly less median intraoperative blood loss and hospital stay in laparoscopic RAMPS [8].

## Conclusions

Herein, we reported a successfully total laparoscopic left-posterior SMA first-approach RAMPS for distal pancreatic cancer in a patient of pancreatic ductal adenocarcinoma with Staging pT2N1M0 with negative margin (R0 resection). There were no short-term complications. We think this technique was safe and effective to perform precise lymphatic dissection in laparoscopic field. Further investigations and follow-up must be proceeded to evaluate the long-term outcomes of our technique.

## Supplementary Information

**Additional file 1: Video 1**. Patient’s information, trocars’ placement, and main steps of our technique.

## Data Availability

Data is available upon reasonable request and with permission of Bach Mai Hospital.

## Abbreviations

AJCC: American Joint Committee on Cancer
CA: Celiac artery
CEA: Carcinoembryonic antigen
CHA: Common hepatic artery
CT: Computed tomography
EUS: Endoscopic ultrasound
FJA: First jejunal artery
FJV: First jejunal vein
GDA: Gastroduodenal artery
IPDA: Inferior pancreaticoduodenal artery
IPDV: Inferior pancreaticoduodenal vein
JPS: Japanese Pancreas Society
LN: Lymph nodes
LRV: Left renal vein
NCCN: National Comprehensive Cancer Network
PDAC: Pancreatic ductal adenocarcinoma
POPF: Postoperative pancreatic fistula
PPDA: Posterior pancreaticoduodenal artery
PD: Pancreaticoduodenectomy
SMA: Superior mesenteric artery
SMV: Superior mesenteric vein
SRPS: Standard retrograde pancreato-splenectomy
RAMPS: Radical antegrade modular pancreato-splenectomy
RGA: Right gastric artery
